# Supplementary material for: A new model of Notch signalling: Control of Notch receptor cis-inhibition via Notch ligand dimers
Source: PLoS Comput Biol. 2023 Jan 20;19(1):e1010169. doi: 10.1371/journal.pcbi.1010169 (PMC9891537; doi:10.1371/journal.pcbi.1010169)
Supplement: S1 Text — The supplementary document provides modeling details, four supplementary figures and MATLAB code for the main text. (DOCX) [file pcbi.1010169.s001.docx]

**Supplementary Information**

**Model with Notch ligand dimerization**

Given any cell containing Notch ligand monomers ($L$), Notch ligand dimers ($L^{*}$), Notch receptors ($N$) and the Notch intracellular domain ($S$), we consider the following protein-protein interactions in the cell:

$\begin{matrix} L+L\underset{\leftrightarrow}{\mathrm{binding}}[LL]\underset{\to}{\mathrm{dimerization}}L^{*}, Ligand dimerization, with relative rate k_{d}. \\ L^{*}+N\underset{\leftrightarrow}{\mathrm{binding}}[L^{*}N]\underset{\to}{\mathrm{inhibition}}\emptyset, Cis-inhibition, with relative rate k_{ci}. \\ \begin{matrix} L_{ext}+N\underset{\leftrightarrow}{\mathrm{binding}}[L_{ext}N]\underset{\to}{\mathrm{cleavage}}S, Trans-activation, with relative rate k_{t}. \\ L+N\underset{\leftrightarrow}{\mathrm{binding}}[LN]\underset{\to}{\mathrm{cleavage}}S, Cis-activation, with relative rate k_{ca}. \end{matrix} \end{matrix}$ (S1)

We assume that all interactions are completed in two steps. The first step is protein complex formation, which is always reversible. The second step forms ligand dimers, blocks Notch receptor activation or leads to Notch receptor activation, that is, the cleavage of the Notch receptor intracellular domain. To express this mathematically, let us consider the dimerization event described in S1. We generate the following ordinary differential equation, which takes account of ligand protein production ($L_{0}$) and degradation ($\beta$):

$\frac{dL}{dt}=L_{0}-\beta L-2k_{d}L^{2},$ (S2)

where $k_{d}$ is dimerization rate of ligand monomers. We use the constant parameters to quantify the relative rates of all interactions. Note that the empty set symbol in the second interaction (S1) means that the Notch receptor is inactivated by ligand dimer.

**A formula for trans-activation in the cells exposed to fixed level of external ligand**

For engineering cells in which the expression of Notch ligand was inhibited ($L_{0}=0$), Eq. (2) and Eq. (7) reduce to

$\left\{ \begin{aligned} \begin{matrix} \frac{dN}{dt}=N_{0}-\beta N-k_{t}L_{ext}N, \end{matrix} \\ \frac{dS}{dt}=k_{t}L_{ext}N-\beta_{S}S, \end{aligned} \right.$ (S3)

where $L_{ext}$ is the level of ligand available for trans-activation of Notch. The non-negative equilibrium ($\bar{N},\bar{S}$) of system (S3) satisfies

$$\begin{matrix} N_{0}-\beta\bar{N}-k_{t}L_{ext}\bar{N}=0, \\ k_{t}L_{ext}\bar{N}-\beta_{S}\bar{S}=0. \end{matrix}$$

Based on these equations, the stable Notch activity is given by

$$\bar{S}=\frac{N_{0}/\beta_{S}}{\beta/k_{t}+L_{ext}}L_{ext}.$$

In experiments, a fluorescent protein related to Notch-activity is often used to quantify the Notch activity since the time series of fluorescence intensity $F\left( t \right)$ can be obtained by tracking living cells. The fluorescent protein is relative stable, so we assume that the fluorescence ($F\left( t \right)$) of cells is proportional to cumulative level of NICD (denoted as $S$). Consequently, $F\left( t \right)=A\int_{0}^{t} S\left( \tau\right)d\tau$, where $A$ is a transfer constant from protein level to fluorescence. Considering that,

$$F\left( t \right)=A\int_{0}^{T} S\left( \tau\right)d\tau+A\int_{T}^{t} S\left( \tau\right)d\tau.$$

When the reactions in a cell reach the stable state ($T$ is relatively big), we have

$F\left( t \right)=A\bar{S}t-A\bar{S}T+A\int_{0}^{T} S\left( \tau\right)d\tau.$ (S4)

Therefore, at the steady state, the production rate of fluorescence is

$R=F^{'}\left( t \right)=A\bar{S}=\frac{AN_{0}/\beta_{S}}{\beta/k_{t}+L_{ext}}L_{ext}$ (S5)

Based on the data on trans-activation obtained by Sprinzak et al. [1], we estimate the parameters in the formula (S5) from 100 iterations of fitting 8 time points, chosen randomly with replacement, out of a total 12 measured time points. The fitting results are in Figs C and D.

References

1. Sprinzak D, Lakhanpal A, LeBon L, Santat LA, Fontes ME, Anderson GA, et al. Cis-interactions between Notch and Delta generate mutually exclusive signalling states. Nature. 2010;465(7294):86--90.

**Code running on MATLAB R2021a (license from Leiden university)**

https://github.com/DaipengChen/A-new-model-of-Notch-signalling


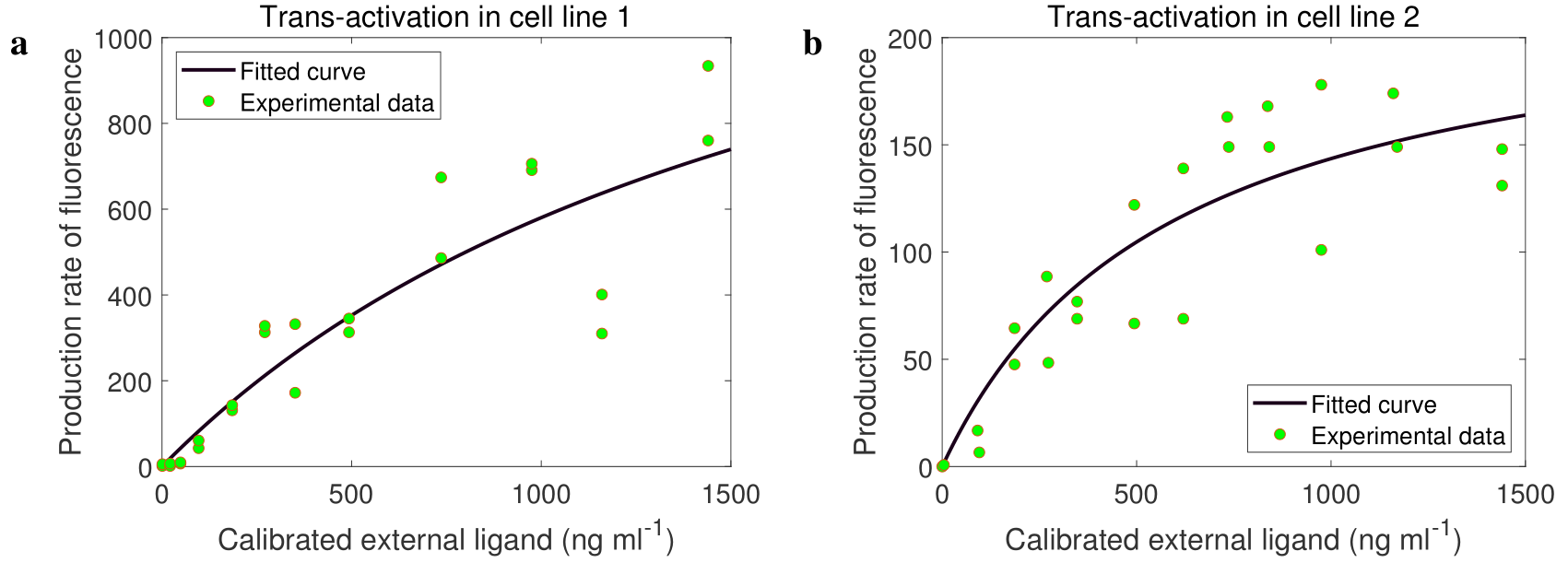


**Fig A.** Testing the general applicability of our model on the dynamics of trans-activation in Notch signaling. (a-b) Eq. S5 was fitted to the observed experimental Notch trans-activation results (1). Here cell line 1 and cell line 2 have different Notch receptor structure. The fitting parameters are two combined parameters $AN_{0}/\beta_{S}$ and $\beta/k_{t}$ in Eq. (S5).


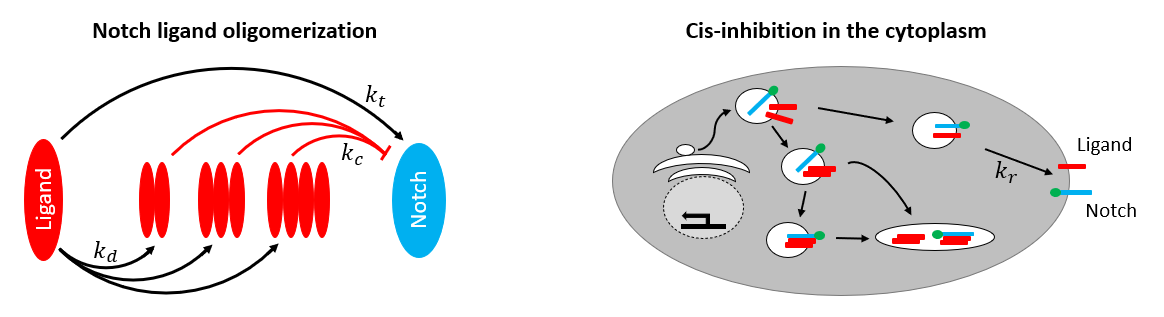
**Fig B.** Schematic representation of Notch ligand oligomerization and the model where ligand dimerization and cis-inhibition of Notch happen in the cytoplasm. Only non-inactivated ligand and receptor can move from the cytoplasm to membrane.


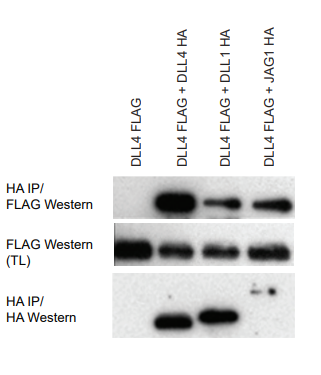


**Fig C.** Notch ligand heterodimerization. The indicated constructs were transfected into tissue-culture cells. Complexes were resolved by immunoprecipitation and visualized with the shown antibodies.


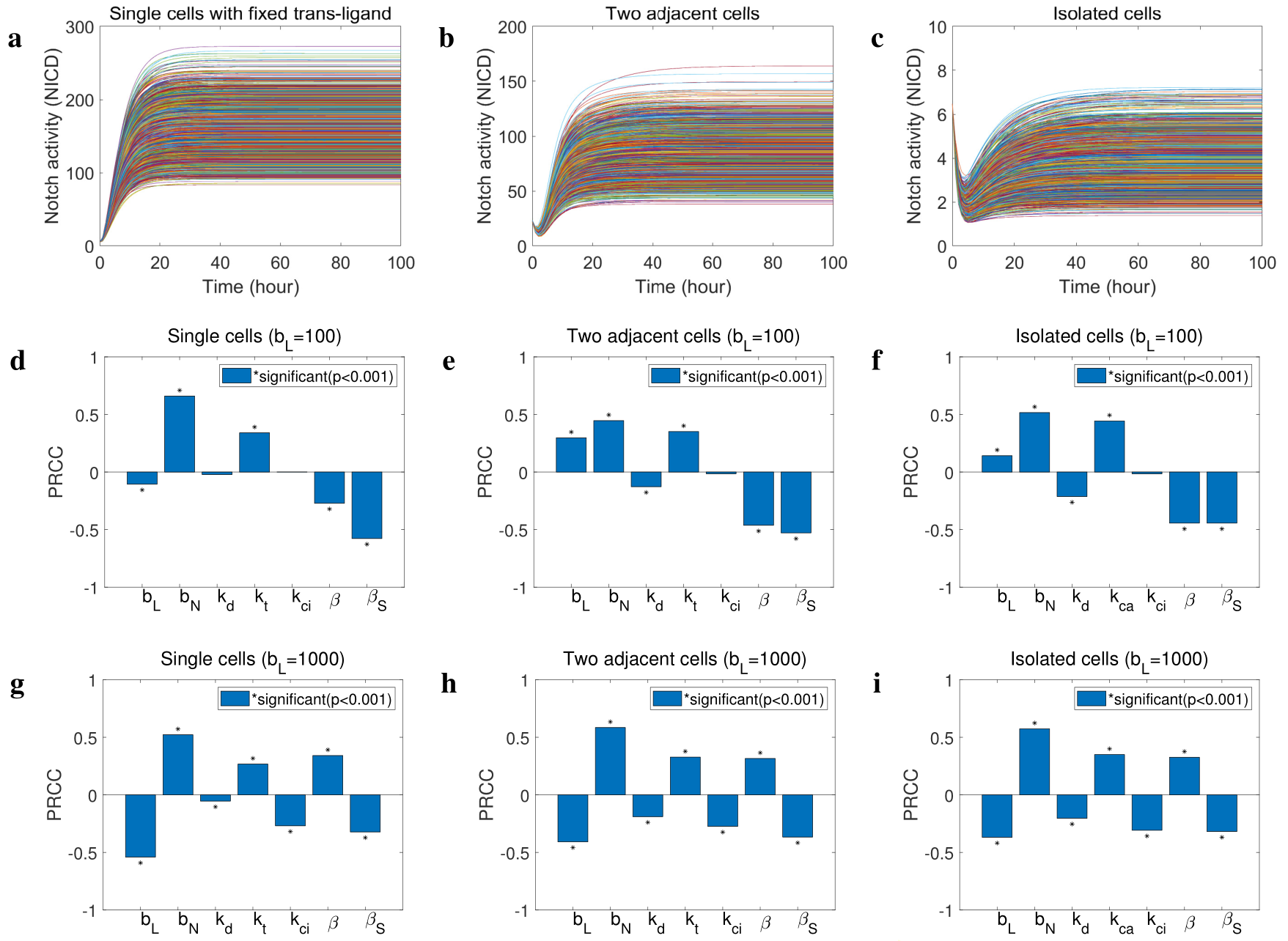


**Fig D.** Parameter sensitivity analysis. The parameter sets are generated using hypercube sampling. Each parameter $par$ is generated from 0 to $2*par$ around the baseline of all parameters (Table 1). (a-c) The simulated time series of Notch activity in single cells exposed to fixed level of external ligands (a), in two interacting cells (b), and in isolated cells (c) using different parameter sets. (d-i) PRCCs for the eight parameters for the steady state of the Notch activity in different simulation settings. Because of the non-monotonic response of Notch to intracellular ligands, we considered low levels of intracellular ligand ($L_{0}=100$) and high levels of intracellular ligand ($L_{0}=1000$).
