# Supplementary material for: A new model of Notch signalling: Control of Notch receptor cis-inhibition via Notch ligand dimers
Source: PLoS Comput Biol. 2023 Jan 20;19(1):e1010169. doi: 10.1371/journal.pcbi.1010169 (PMC9891537; doi:10.1371/journal.pcbi.1010169)
Supplement: Attachment — Submitted filename: Chen et al. Revised manuscript Cover letter 19.09.22.docx [file pcbi.1010169.s004.docx]

**David Baker PhD & Roeland Merks PhD**
Mathematical Institute, Leiden University
& Department of Cell & Chemical Biology,

Leiden University Medical Center (LUMC)
2300 RC Leiden
The Netherlands
email: [d.baker@lumc.nl](mailto:d.baker@lumc.nl)

merksrmh@math.leidenuniv.nl
Tel: +31 71 526 9223

+31 71 527 7106

Wednesday 21^st^ September, 2022.

Dear Professors Meier-Schellersheim & Haugh,

We would be most grateful if you would consider the accompanying revised manuscript for publication as an Article in PLOS Computational Biology. We were delighted that the reviewers were generally very supportive of our work and we thank the reviewers for their helpful suggestions for amending our manuscript. We have comprehensively addressed these issues yielding, we believe, a substantially improved manuscript. Specifically, we have clarified the details of the experimental work and updated the figures to more clearly demonstrate cis inhibition in the context of trans activation stimulated by ligand expressed in neighbouring cells. We present and explain in detail a general equation and describe in depth how our model relates to relevant existing models. We have performed a number of new simulations at the molecular and cell level, which consolidate our earlier findings and unveil how our model can recapitulate published experimental findings. We further extend these analyses by demonstrating that simulations using our model can reproduce *in vivo* tissue patterning processes (*Drosophila* wing morphogenesis), which have presented a challenge to other models.

We remain confident that our work will be of broad appeal to both the scientific and clinical community across multiple disciplines including cell biology, physiology and systems biology, and pathologies thereof.

We very much hope that our manuscript is now suitable for publication. Should you require any further information, please do not hesitate to contact me.

Our response to each of the reviewers’ remarks are detailed below. Two versions of the manuscript are provided: an unmarked version and one in which changes have been highlighted.

We look forward to hearing from you.

Yours sincerely,

David Baker

Roeland Merks

*Mathematical Institute, Leiden University &*

*Leiden University Medical Center (LUMC)
Leiden
The Netherlands.
Email:* [d.baker@lumc.nl](mailto:d.baker@lumc.nl)

[merksrmh@math.leidenuniv.nl](mailto:merksrmh@math.leidenuniv.nl)

**REVIEWER 1**

The reviewer raised a number of important points related to the experimental work and modelling. The reviewer did remark perhaps we had ‘missed an opportunity here to identify differences that are substantial and that can be tested experimentally (even if not within this manuscript)’.

**Response:** We have addressed each remark in turn either through necessary clarifications and/or by additional analyses. Moreover, we present a completely new modelling figure (Fig 9) in which the ligand dimerization model, but not other models, is shown to reproduce the role of Notch signalling in *in vivo* tissue patterning.

**The Biochemical data in Fig. 1-3 show that Dll4 and Dll1 ligands can multimerize and that the interacting domains are EGF8 and MNNL. This is used in Fig. 4 to show that Dll4 lacking the EGF8 can trans-activate but not cis-inhibit. This is an important observation, however the assay used to make this observation is unclear:
a. The information provided in the text, captions, and methods is sketchy with many details missing. First, for measuring cis-inhibition one needs to provide separately ligand in-trans (plate-bound-Delta of co-cultures Delta cells) and in-cis (expression in the same cell). From the description of Fig. 4A it is not clear that this was done, as there is no trans-Delta provided. These experiments should be performed with trans-Delta. Moreover, it should be shown that this signal is Notch dependent for example by blocking gamma-secretase.**

**Response:** The reviewer is correct that our description/presentation of the data shown in Fig 4 was indeed rather cursory. In part this stemmed from our decision to keep the experimental part of the paper as succinct as possible to enable a greater focus on our modelling. Indeed this point, regarding the relative emphasis on experimental vs modelling data, was raised by reviewer 3. Cis inhibition assays were performed in a variety ways, using both transitory transfection approaches as well as using engineered stable cell lines ectopically expressing appropriate combinations of Notch receptors and ligands (yielding comparable results), and one of these approaches is now shown. Trans ligand was provided in these assays, and this is explained in more detail both in the Methods and also the figure legend. Since the precise mechanistic nature of cis inhibition remains to be fully elucidated, in these cases, we expressly avoided using plated recombinant ligand and instead used ligand stably expressed in cells (and which thus undergoes normal trafficking, post-translational modifications, processing etc.). Interestingly, through development of a novel method, one of our related projects is to purify (to homogeneity) Notch receptors and ligands from human cells in sufficient quantities to enable more detailed architectural/structural studies. Compellingly, our initial studies (thus far with Dll1) show that it is indeed dimeric.

**b. More information is needed on the co-culture experiments including how long prior to co-culture where the cells transfected, what was transfection method and efficiency, how were the cells moves to the co-culture dish and so on. Also, here DAPT inhibition should be provided. Also, a control where non-activating ligand is used (DeltaEGF1-3 or similar) should be added.**

**Response:** We have now provided additional details of our assays and included an analysis in the presence and absence of DAPT, which together with our own mutational analysis of DLL4, demonstrate dependence on Notch activation.

**c. It is not mentioned what the error bars represents – whether the error is between repeats? Where the triplicates performed in different days? There is no statistical analysis of the results check significance?**

**Response:** Each data point represents the mean of three independent readings and the error bars show the standard deviation. Experiments were performed at different times at least 3 times, either transitory transfections or separate lentiviral infections, and representative experiments are shown with associated Notch ligand and receptor protein levels determined. We have updated the legend of Fig 4 and provide more details in the Methods section (lines 555-560).

**2. It has been previously shown in Drosophila that Serrate lacking EGF repeat 4,5, or 6 can also trans-activate (in vivo) but do not cis-inhibit (Fleming 2013, PMID: 23571220). This should of course be mentioned in the manuscript. The fact that similar deletion can also remove cis-inhibition in mammalian Dll1/4 is an interesting observation that generalize the results in the manuscript.**

**Response:** We agree with the reviewer that this is indeed a very interesting finding, which we have now referenced in the manuscript. Moreover, we have performed simulations to recapitulate some of the experiments described in this article (shown in Fig 9 and discussed in lines 358-412).

**3. Regarding the modeling. As I mentioned before the suggested equations (eq. 1-2,3) makes sense for describing the cis-inhibition mediated by ligand dimerization. However, the explanations on the different aspects of the models are severely lacking, and the conclusions drawn are either exaggerated or not substantiated. These include:
a. In Fig. 4A-C the description of what is calculated and how is unclear. It not clear which Notch activity is calculated (from which of the two cells) and whether the parameters change only in one cell or in both. A better** **Scheme showing the model setup of two cells would help.**

**Response:** The reviewer is correct. To address this, we have added more detailed descriptions in lines 236-238 and in the legend of Fig 6 (revised version) to clarify what is calculated and how the calculation is performed. We have also followed the reviewer’s advice and now present schematic representations of the model setup.

**b. In line 214 there are misleading statements regarding the choice of parameter. it is said that: “…the ligand dimerization rate, based upon previously published estimates”. This is clearly not the case as Table 1 shows that this rate and other rates are assumed (just guessed). This is mentioned in other places (discussion) and it should be explained where this and other parameters come from.**

**Response:** We have removed these ambiguous statements and have added details in the Methods section (lines 490-499), which explain more clearly the use of and the nature of the parameters.

**c. The degradation rate of the dimer is assumed to be 10 time slower than the monomers. There is no explanation why this is assumed (no experimental data). Moreover, it should be discussed whether this difference is essential for the results presented.**

**Response:** The reviewer is correct that we do not have data to support this assumption, however, the modelling results are not dependent on it. We have removed this assumption and updated all simulations.

**d. At the end of the paragraph it is said that “the mathematical model (1), describing ligand-oligomer-specific cis inhibition of Notch signalling, faithfully recapitulates our experimental findings”. It is really unclear which finding are referred to since in the experiments in Fig. 4 Ligand concentration and kd were not varied!**

**Response:** The reviewer is correct that the ligand dimerization rate is not a continuous variable in experiments. Because we do not know the exact value of the dimerization rate of WT ligands and mutant ligands in cells, we focused on the response of Notch activity in cells to varying ligand dimerization rates when the level of cis-ligand is high in Fig 6C. We have clarified our statements in main text (see lines 240-246).

**e. The only non-trivial observation in Fig. 4 is that dimer deficient ligands trans-activate but do not cis-inhibit. So, the authors should consider simulating this aspect i.e., comparing WT and mutant ligand behaviors in different models. Moreover, the models that should be compared here are the model that dimerization drive cis-inhibition (case 1) and the old model where monomers drive cis-inhibition. One could add a model where both monomers and dimers exert cis-inhibition.**

**Response:** We agree with the reviewer and have addressed each of these points in Fig 6. Specifically, we do consider monomer, dimer and monomer/dimer- driven cis inhibition (lines 231-234 and Fig 6). Moreover, as mentioned earlier, we have compared the dimer and monomer models by considering *in vivo* tissue-patterning in Drosophila wing morphogenesis (Fig 9 and lines 358-412).

**f. Instead of simulating two cells, it might be better to simulate only a single cell that feels some external ligand level, and show Notch activity as a function of the cis ligand level. Then one can show this plot for different models as described in e.**

**Response:** We have simulated a single cell exposed to fixed trans ligand level for different models in Fig 6A-6C (descriptions in lines 236-238).

**g. In Figure 6 the authors compare the ability of their model to explain results obtained in ref 22. They show that their mode can qualitatively capture the experimental results obtained. However, they also claim that “…it appears to more closely match the observed pattern of ligand decay”. This statement is not substantiated by any measure and should either be proven (statistically) or removed. Moreover, claiming that your model is better than the old model cannot be based on small quantitative differences that may simply reflect the choice of parameters and can be easily tuned in either model. To distinguish between models, one should bring a suggested experiment that significant qualitative difference between the two models.**

**Response:** Indeed, we agree with the reviewer that the pattern of ligand decay depends on parameters. We have removed the unsubstantiated remarks and corrected our statements related to this point (lines 272-274).

**h. In Fig. 6C, D it is completely unclear which data is being used to perform the fit (this is again from ref 22, but cell line 1 and cell line 2 are not defined). The meaning of these fits is pointless as the original data simply show a sigmoidal dependence on external ligand without cis-inhibition (only plate bound Delta). So, there is no meaning to claiming anything regarding the new model.**

**Response:** The reviewer is correct that there is no difference between our model and old model for Notch receptor trans-activation mediated by fixed external ligands. Ostensibly, we performed these analyses to demonstrate that the dimer model could reproduce previously published work, rather than to explicitly highlight differences between the models (lines 272-274). We have removed these two figures from the main body of the paper (old Fig 6 C & D; now presented as SI).

**i. In Fig. 7 there is a comparison of different models that include cis-activation. Here, they authors claim that their model can explain better the results from ref. 31. Once again, the claim is not substantiated. The claim is: “Notably, our ligand dimerization model generates narrower peaks using the same reference parameter values, which is closer to experimental data”. This claim has no meaning as it does not really matter what is the width of the peaks obtained in a specific realization of the simulation since it can be changed by changing the parameters. A better approach would have been to discuss the prediction of an experiment using the dimerization deficient ligand (this is what is approximately shown in Fig. 7G.**

**Response:** The reviewer is correct that the width of the peaks is parameters-dependent. We have removed this statement and as recommended provided a more in-depth discussion of modelling predictions presented in Fig 7H and 7G (see lines 315-319).

**j. Fig. 8 addresses the comparison between different levels of oligomerization. It is again unclear what is plotted in Fig. 8B and how the model is compared to experiments. This is also true for Fig. 8D.**

**Response:** We have updated Fig 8 and have provided more information in the legend and main text (lines 334-338).

**k. It is not clear why data is discussed for the first time in the discussion (models S3, S4). It should be moved to the results.**

**Response:** The results have been moved to Fig 8 of the Results section and are now discussed in this section (lines 351-356).

**l. Along the lines in the discussion it is claimed that: “we consistently observed enhanced reporter activity under cis conditions in the presence of DLL4 ligand monomer but not DLL4 ligand dimer, which instead strongly inhibited Notch receptor activity”. This is not discussed in the results nor is it shown that this difference is statistically significance.**

**Response:** The reviewer is correct that the statement is both vague and unsubstantiated, and has now been deleted.

**m. Line 316 in the discussion: “Using physiologically relevant parameters our simulations strongly favour the former case”. This is not a substantiated statement.**

**Response:** We have corrected this statement in lines 438-439.

**Minor concerns:
4. In Fig. 2 the western and commassie staining has poor resolution (hard to read the text).**

**Response:** We have modified this Figure.

**5. Fig. 3B has a different font than Fig. 3A**

**Response:** The font has been corrected.

**6. Resolution of the images in Fig. 4B is low. It is not explained what is calnexin and why is it stained**

**Response:** The resolution has been adjusted and an explanation for staining is provided.

**7. The full code should be uploaded to a github.**

**Response:** All relevant code will be uploaded.

**REVIEWER 2**

The reviewer raised four specific points related to mathematical modelling and offered excellent advice. We have addressed each point in turn either through necessary clarifications and/or by additional analyses.

**1.The discussion about different proposed models is very confusing. Firstly, the Sprinzak equations are frequently used and referred to in the text. They turn out to be Equations (5) of the Methods section. It would be helpful to write them before, in the Results section, and refer to them explicitly by number when needed. Otherwise, it seems one has to keep reading Sprinzak paper in parallel to this one to follow the authors’ arguments, until it is discovered that the Sprinzak equations are listed as (5) later in the manuscript.**

**Response:** We agree with the reviewer. We have moved the Sprinzak equations to the results section (Eq. 2), explained the relevant details more fully (lines 204-209), and now consider it in detail (lines 210-220) alongside our ligand dimerization model (lines 280-290).

**Secondly, it would be helpful if the authors make clear which of their models is best for the general case, including the contributions ‘ext’ in Eq. (4) if appropriate. One possibility would be writing a general equation instead of (2) and (4) that** **comprises all terms in these equations, and then specifying which terms should be set equal to zero according to which experimental information and situation.**

**Response:** This is excellent advice. We have now written the general equations (1) that comprises all relevant terms, which are now described in detail for extra clarity (lines 183-196).

**2. The papers by Boareto et al are referred to but not really discussed. Boareto et al studied two coupled cells with equations similar to Sprinzak’s and defined sender/receiver (or tip/stalk) phenotypes. By adding Jagged dynamics to the Sprinzak equations, Boareto et al discovered new hybrid sender/receiver (tip/stalk) cellular phenotypes that turn out to play a role in sprouting and tumor angiogenesis and can be incorporated to cellular models of angiogenesis; see Vega et al, PLoS Comp. Biol. 16, e1006919, 2020. Hybrid phenotypes can be conveniently extended as tumor transition states during EMT, which have been experimentally identified by Pastushenko et al, Nature 556, 463 (2018). The present authors should comment on the consequences for hybrid sender/receiver phenotypes that possible extensions of their models to include Jagged dynamics could have.**

**Response:** The reviewer is correct. We have extended our discussion of these papers.

**3. Eq. (1) considers only one type of ligand oligomers of size n, and then the authors make the case that n=2. Suppose that there are concurring oligomerization processes with n=2 and higher. Would it be possible to** **discriminate these processes and reach the** **same conclusion about n=2?**

**Response:** We thank the reviewer for this remark. In Fig 8, we consider oligomers of different size (up to n = 4), and find that the outcome is independent of oligomer size within this range (lines 334-338).

**REVIEWER 3**

The reviewer pointed out that the paper is heavily focused on the experimental work, rather than computational biology and expected to see the balance more heavily focused on the theoretical aspects for PLOS Computational Biology.

**Response:** We agree with the reviewer. We have resolved to keep our experimental results as concise as possible whilst extending our modelling analyses (lines 210-220 and 280-290), specifically by performing additional simulations including a completely new modelling analysis of *in vivo* tissue patterning (lines 358-412), shown by Fig 9.

**• The literature review is rather brief, and could be expanded to include a description of biological applications of delta-notch signalling (e.g., angiogenesis, cell-fate specification in the intestinal crypt) and theoretical studies of these systems. This would provide stronger motivation for the authors’ research.**

**Response:** The reviewer is correct. We have added additional information both in the introduction and also in the results section (by way of introduction to our modelling tissue morphogenesis).

**• It would also be helpful if the authors explained some of the technical terminology and clarify the extent to which the detailed biological results map onto, and motivate, the mathematical models that follow. In particular, is it possible to measure the number of ligands involved in cis inhibition and trans activation and/or the number of monomers, dimers, trimers, etc. on a particular cell?**

**Response:** We agree with the reviewer. We have added some diagrammatic representations of Notch signalling in Figs 5 and 6 to help readers better understand the mathematical models. Regarding ligand ‘architecture’ and dynamics, currently, it is impossible to measure the number of ligands involved in cis inhibition and trans activation and/or the relative numbers of monomers, dimers, trimers, experimentally. We do show, in Fig 8, that our modelling predictions would not be changed by different oligomer sizes (lines 334-338).

**• It would be easier to understand and interpret the experimental results if they clarified (in figure captions, e.g., Fig1, or in Methods) which antibody is for what and how to interpret their results****.**

**Response:** In the methods section, we have added additional information about the antibodies.

**• Please clarify how the experimental results (e.g., Fig 4A) are used. Are the data fitted to the models, to estimate the model parameters (e.g., k_{cis})?**

**Response:** In Fig 6B-C and 6E-F, we have clarified the nature of the correspondence between the numerical simulations and the experimental results (lines 240-246). Regarding parameters, because of over-fitting, it is not possible to use the data to estimate model parameters.

**• Please improve the quality of the images in Fig 2.**

**Response:** An adapted Fig 2 is now presented.

**• Lines 133-134: What is the difference between the results obtained from tissue culture cells and in vitro?**

**Response:** Essentially, results obtained from tissue culture cells relates to ligands expressed in cells, which means that the ligand interactions occurred in the cell. By contrast, *in vitro* experiments utilize purified recombinant ligand proteins. Such interactions are, in principle, more direct since they occur in the absence of other cellular proteins.

**• Equations (1) – it would be helpful to clarify earlier the context in which these equations are relevant – are they relevant to an assumed homogeneous population of cells or not (this becomes clear later)? Also, please clarify that n = oligomer size, and state clearly that oligomers can only cis-inhibit. Please also explain why you do not consider monomers, dimers, trimers, etc. (i.e., a sequence of binding complexes) rather than just n ligands.**

**Response:** We have clarified that the general Eq. (1) describes ligand/receptor interactions in any given cell (lines 183-184). The equation is valid for a homogeneous population of cells (Fig 6G-H), and we now also consider heterogeneous populations of cells (Fig 9). In both instances, the model reproduces known experimental findings.

We now clarify that n = oligomer size and that indeed only oligomers can execute cis inhibition (Fig 6C and lines 247-248). We agree with the reviewer (a point also raised by reviewer 2). We now provide the necessary clarifications earlier in the main text (lines 183-196). Finally, in Fig 8, we clarify and explain the nature of oligomer size (lines 334-338).

**• It would be helpful if the authors included a schematic in the main text distinguishing the different models that they study, especially Sprinzak’s.**

**Response:** We agree with the reviewer (a point also raised by reviewer 2). New schematics are now provided both in Fig 5 and Fig 6.

**• Line 236: where do the authors show that the model with dimerization more closely matches** **the observed pattern of ligand decay? Where is the comparison between the data and other modelling assumptions (eg monomer, trimer, …)? Where is the comparison with the fit of Sprinzak’s model to the data?**

**Response:** The issue of ligand decay was also raised by Reviewer 1. It is correct to say that the pattern of ligand decay is indeed parameter-dependent, and thus cannot quantitatively distinguish between models in a meaningful way. We have removed the unsubstantiated remarks and corrected our statements related to this point (lines 272-274).

**• [related to** **above comment] Fig 6. Please clarify what is being measured, and explain why your fitting is better than that of Sprinzak. How was the model fitted to the data? What are the error bars (and confidence intervals) on your parameter estimates?**

**Response:** Please see response above. To distinguish between models, we have considered ligand dimerization rates in 2 cells (Fig 6C and 6F), and later in populations of cells (Fig 9), which demonstrate clear differences between models.

**• Figure 6(C): it would be helpful to include results for other models, involving monomers, trimers and tetramers, to explain why dimers are found to give the best agreement with the experimental data. It would also be helpful to understand the implications of distinguishing monomeric activation and dimeric inhibition on Notch signalling dynamics – could the authors suggest experiments which clarify this? How important is distinguishing monomeric and dimeric binding in comparison with stochastic effects, including noise?**

**Response:** In Fig 6C we previously showed that our model, in common with other models, including the Sprinzak model, could reproduce experimentally-derived Notch trans activation data. Thus, for this trans activation, there is no difference between our dimer model and the other monomer-centered models. Indeed, we performed these analyses to demonstrate that the dimer model could reproduce previously published work, rather than to explicitly highlight differences between the models. As alluded to earlier, these differences become evident when we consider cis inhibition. We have now considered multiple different scenarios (involving ligand monomers and dimers) for both trans activation and cis inhibition, shown in Fig 6. We further clarify the significant differences between our dimer model and other monomer-based models by considering simulations of *in vivo* tissue patterning (only the dimer model can fully reproduce this- see Fig 9).

**• To what extent are the results in Figure 8 based on model fits to the experimental data? Where are the fitted parameter values and confidence intervals? Is it possible that there is a combination of monomers, dimers, trimers, etc?**

**Response:** In this instance, we did not fit our mathematical model to the experimental data since only monomers and oligomers were tested (and it is currently not possible to precisely elucidate the size of ligand oligomers). We do now consider oligomers of different size (up to n = 4) and a combination of oligomers (lines 331-334), and find that the outcome is independent of oligomer size within this range (Fig 8A-8C and lines 334-338).

**• Table 1 – the majority of the parameters are estimated from ref [27] – could the authors clarify** **how the estimates were obtained – in the original article? I note also that refs [26] and [28] relate to theoretical models – could the authors clarify the extent to which their parameters were estimated by fitting their model to experimental data?**

**Response:** In the Methods section, we have further explained the parameter sources (lines 490-499) and have clarified how the estimates were obtained in the original papers [26,27,28].
