# Supplementary material for: A new model of Notch signalling: Control of Notch receptor cis-inhibition via Notch ligand dimers
Source: PLoS Comput Biol. 2023 Jan 20;19(1):e1010169. doi: 10.1371/journal.pcbi.1010169 (PMC9891537; doi:10.1371/journal.pcbi.1010169)
Supplement: Attachment — Submitted filename: Chen et al. Revised Manuscript Coverletter 27.11.22.docx [file pcbi.1010169.s005.docx]

**David Baker PhD & Roeland Merks PhD**
Mathematical Institute, Leiden University
& Department of Cell & Chemical Biology,

Leiden University Medical Center (LUMC)
2300 RC Leiden
The Netherlands
email: [d.baker@lumc.nl](mailto:d.baker@lumc.nl)

merksrmh@math.leidenuniv.nl
Tel: +31 71 526 9223

+31 71 527 7106

Wednesday 23^rd^ November, 2022.

Dear Professors Meier-Schellersheim & Haugh,

We would be most grateful if you would consider the accompanying revised manuscript for publication as an Article in PLOS Computational Biology. We were delighted that the reviewers are very supportive of our work and are satisfied that our manuscript has been significantly improved- we thank the reviewers for their helpful suggestions for further amending our manuscript. The reviewers raised a small number of ‘editorial’ issues/questions, which we have addressed in the comments below and the accompanying manuscript.

We remain confident that our work will be of broad appeal to both the scientific and clinical community across multiple disciplines including cell biology, physiology and systems biology, and pathologies thereof.

We very much hope that our manuscript is now suitable for publication. Should you require any further information, please do not hesitate to contact me.

Our response to each of the reviewers’ remarks are detailed below. Two versions of the manuscript are provided: an unmarked version and one in which changes have been highlighted.

We look forward to hearing from you.

Yours sincerely,

David Baker

Roeland Merks

*Mathematical Institute, Leiden University &*

*Leiden University Medical Center (LUMC)
Leiden
The Netherlands.
Email:* [d.baker@lumc.nl](mailto:d.baker@lumc.nl)

[merksrmh@math.leidenuniv.nl](mailto:merksrmh@math.leidenuniv.nl)

**REVIEWER 1**

**Regarding figure 4 – I think it would add if a schematic of the co-culture experiments of Fig. 4A would be included. In the Cis(+DAPT) panel, it should be explained what Notch (-) refers to. Finally, p-values for significance should be included in the caption.**

**Response:** The reviewer is correct. We have added a schematic of the co-culture experiments in Fig. 4A and have explained what Notch (-) refers to in the caption of Figure 4. We have also added p-values for significance in the caption.

**The Authors show in Fig.4 that ligands that lack EGF repeats does not cis-inhibit Notch2. Have they tested also the effect on Notch1? If they have, it would be a nice addition. If not, it is sufficient to mention in the discussion whether they think it would be having the same with different Notches.**

**Response:** This is indeed a very valid point and is something we are currently investigating, as well as a more detailed characterization of the other vertebrate Notch ligands (lines 485-490). However, at this time, in terms of data presentation, this will be better considered in another manuscript.

**When discussing Figure 6B it should be mentioned that** **the reduction in Notch activity decreases slower in model C1 (cis-inhibition through dimerization) compared to the other models. This is something that can be tested experimentally.**

**Response:** This is a good point. We have discussed the reduction of Notch activity predicted by model C1 and other models in lines 240-243.

**In Figure 6I the authors show that their model can reproduce the experimental result in the Sprinzak et al paper. It should be noted that also the regular cis-inhibition model matched the experiments. Namely, that the new model in this case is consistent with the experimental results as does the previous model. Hence, the new model does not explain a previously unaccounted for phenomena.**

**Response:** The reviewer is correct. We have clarified that our new model is consistent with the mutual inactivation model in explaining these experimental data in lines 279-280.

**The steady analysis for the new model and its comparison with the old model is quite revealing. One important aspect that comes out of this analysis is that even when Notch levels are high the monomer ligand is free to activate neighboring cells. This is a key difference that its implication can be studied experimentally as the authors try to do in Figure 9.**

**Response:** This is an excellent point. We have discussed the key difference in lines 503-506.

**When describing the model where cis-inhibition happen in the cytoplasm, the authors should explicitly mention that only** **non-cis-inhibited ligands/receptors can go from the cytoplasm to the membrane. A diagram showing the assumptions of this model would be useful.**

**Response:** This is indeed a valid point. To address it, we have mentioned that only non-cis-inhibited ligands/receptors can go from the cytoplasm to the membrane in lines 353-354. We have added a diagram in Fig SI 2 and mentioned it in line 354.

**The conclusion of the analysis of that model in the text states that: “These simulations demonstrate that the modelling predictions do not depend on the subcellular localization of ligand dimerization and dimer mediated cis-inhibition of Notch.” I agree that the qualitative behavior is the same but the quantitative one seem to be different. Suggest to amend statement.**

**Response:** The reviewer is correct. We have amended the statement in line 360-361.

**The new figure 9 is a nice addition to the manuscript but it lacks proper description of the assumptions taken. It should be added in the text and methods the exact assumptions and parameter values considered for each simulation.**

**Response:** The reviewer is correct. We have added more description of the modelling assumptions in lines 392, 395-397 and added the exact values of parameters for each simulation in lines 540-545.

**Regarding the Fig. 9A-B, the authors show that their model exhibit the expected expression pattern of Notch activity. However, is there a difference** **between the mutual inactivation model and the new model? If not, this should be clearly stated.**

**Response:** We agree with the reviewer. We have clarified that there is no difference between the mutual inactivation model and our new model regarding wing vein patterning in lines 382-383. The difference between the two models becomes apparent when we consider dorsal/ventral patterning of the wing.

**Fig. 9C-H argue that the new model can explain the pattern in the wing DV boundary while the mutual inactivation model cannot. I suspect that this depends on the assumptions taken. For example, I believe that mutual inactivation model can explain the observations if cis-inhibition strength is assumed to depend on Fringe activity (as was shown by** **Lebon et al eLife 2014). Moreover, the authors assume that in this case Ligand expression levels across the wing are below Notch expression levels. If it is assumed the opposite (that ligand levels are above Notch levels - I don’t think it is clear what is the case for the real system) can the observed Notch activity distinguish between the two models? This assumption and tis implication should be discussed.**

**Response:** This is a good point. Indeed, Lebon et al. proposed a model to explain DV patterning. But they did not provide numerical simulations to support their hypothesis. Fringe-dependent cis-inhibition reduced the mutual exclusion between Serrate and Notch in dorsal cells but Delta and Notch are still mutually exclusive in ventral cells. We have discussed the work of Lebon et al in lines 407-410. We have also discussed in lines 395-397 that it is reasonable that ligand expression levels across the wing remain below Notch expression levels. In Fig 9E, we have further shown that when Notch receptor levels fall below ligand expression levels we no longer see robust activation of Notch at the DV boundary, thus there is no difference between the wo models under these conditions (see lines 414-416).

**Please note that while Ser is expressed only in the Dorsal, Delta is expresses everywhere. It is more strongly active in the ventral section where fringe is not expressed. Please amend description.**

**Response:** The reviewer is correct. We have amended the description in line 392.

**The patterns of overexpression of Delta and Ser predicted by the model (Fig. 9F-G) do not seem to match the experimental ones since in the experiments the two peaks of expression persist both on the ventral and the dorsal ones. See for example De Celis and Bray, Development 1997. Please explain discrepancy.**

**Response:** We thank the reviewer for highlighting this. This paper has been referenced in the manuscript (see 53 in reference list). Our simulations in Figs 9F-G are consistent with figure 7 and figure 8 in this paper. We have also cited other papers (52-55) as supporting evidence to validate our simulations.

**REVIEWER 2**

In their prior assessment, the reviewer was very positive about our work and raised four specific points (all helpful) related to mathematical modelling and offered excellent advice. We had hoped that we’d satisfactorily addressed each point in turn either through necessary clarifications and/or by additional analyses. We are therefore, sorry that the reviewer feels that we have not sufficiently discussed the papers of Boareto et al.

**Response:** We thank the reviewer for pointing this out. We have added more discussion to Boareto’s papers in lines 492-498, which we hope sufficiently addresses the reviewer’s concerns.

**REVIEWER 3**

**The authors have done a great job of addressing my comments, and their revised manuscript represents a significant improvement over the original submission. In particular, the readability of the theoretical aspects of their work has been improved by moving the statement of their extension to Sprinzak et al’s model into the results section. Also, by softening some of the statements made in the original manuscript the authors provide a more measured account of the impact of their research. I have only two minor comments to raise (see below).**

**Response:** We thank the reviewer for their remarks.

Minor Comments

**Regarding the extent to which their model is consistent with published experimental data (see lines 272-274), it would be helpful if the authors could include a statement clarifying how agreement was quantified and clarifying that they were not able to parameterise their model to experimental data. On a related note, have the authors considered performing a comprehensive parameter sensitivity analysis of their model – this might be helpful, especially with experimental design.**

**Response:** This is an excellent point. We have clarified how agreement (between the model and experimental findings) was quantified and clarified that it is not possible to parameterise the model, in lines 275-277. As suggested, we have performed a parameter sensitivity analysis (see lines 524-527 and Fig SI 4).

**It is confusing to use L and N to denote the number of Notch ligand and receptors per cell and then to use L0 and N0 to denote by production rates, as these quantities have different dimensions. It would be more natural (and less confusing) to denote by L0/tau and N0/tau the rates at which Notch ligand and receptors are produced.**

**Response:** The reviewer is correct. We have appropriately adapted the symbols.
